# Genotype and phenotype analysis of Taiwanese patients with osteogenesis imperfecta

**DOI:** 10.1186/s13023-015-0370-2

**Published:** 2015-12-01

**Authors:** Hsiang-Yu Lin, Chih-Kuang Chuang, Yi-Ning Su, Ming-Ren Chen, Hui-Chin Chiu, Dau-Ming Niu, Shuan-Pei Lin

**Affiliations:** Department of Medicine, Mackay Medical College, New Taipei City, Taiwan; Department of Pediatrics, Mackay Memorial Hospital, No. 92, Sec. 2, Chung-Shan North Road, Taipei, 10449 Taiwan; Department of Medical Research, Mackay Memorial Hospital, Taipei, Taiwan; Mackay Medicine, Nursing and Management College, Taipei, Taiwan; Institute of Clinical Medicine, National Yang-Ming University, Taipei, Taiwan; Medical College, Fu-Jen Catholic University, Taipei, Taiwan; Institute of Biotechnology, National Taipei University of Technology, Taipei, Taiwan; Department of Obstetrics and Gynecology, School of Medicine, College of Medicine, Taipei Medical University, Taipei, Taiwan; Department of Pediatrics, Taipei Veterans General Hospital, Taipei, Taiwan; Department of Infant and Child Care, National Taipei University of Nursing and Health Sciences, Taipei, Taiwan

**Keywords:** Bone mineral density, Genotype, Height, Osteogenesis imperfecta, Phenotype

## Abstract

**Background:**

Osteogenesis imperfecta (OI) is a congenital disorder characterized by increased bone fragility and low bone mass.

**Methods:**

The presence of *COL1A1* or *COL1A2* mutation was investigated by direct sequencing in 72 patients with OI type I, III, or IV (27 males and 45 females; age range 0.2-62 years) from 37 unrelated families. The clinical features of these patients were also recorded.

**Results:**

Thirty-seven *COL1A1* and *COL1A2* mutations were identified, including 28 *COL1A1* mutations and 9 *COL1A2* mutations. Fifteen (41 %) were novel mutations, and twelve (32 %) were familial mutations. A review of their medical records revealed that the 72 patients could be classified into OI type I (*n* = 42), III (*n* = 5), and IV (*n* = 25). Twenty-nine patients had helical mutations (caused by the substitution of a glycine within the Gly-X-Y triplet domain of the triple helix), and 42 had haploinsufficiency mutations (caused by frameshift, nonsense, and splice-site mutations). Compared with haploinsufficiency, the patients with helical mutations had more severely impaired skeletal phenotypes, including shorter height, lower bone mineral density, poorer walking ability, more frequent manifestations of dentinogenesis imperfecta and scoliosis (*p* < 0.05).

**Conclusions:**

Genotype and phenotype databases are expected to promote better genetic counseling and medical care of patients with OI.

**Electronic supplementary material:**

The online version of this article (doi:10.1186/s13023-015-0370-2) contains supplementary material, which is available to authorized users.

## Background

Osteogenesis imperfecta (OI) (MIM# 166200, 166210, 259420, and 166220) is a hereditary disease characterized by increased bone fragility, low bone mass, short stature, and other connective tissue manifestations, with a reported incidence of 1:15,000-1:25,000 [1, 2]. Additional extra-skeletal features manifest to a variable degree, including blue sclera, dentinogenesis imperfecta (DI), and hearing loss [3, 4]. In Western populations, OI is generally inherited in an autosomal dominant manner, however, a small number of families inherit their OI in an autosomal recessive pattern [3]. The most commonly used classification is that proposed by Sillence et al. [5], but as modified and expanded by the International Nomenclature Committee for Constitutional Disorders of the skeleton as reported by van Dijk and Sillence 2014 [3] which classifies OI into five major groups of disorders as types 1–5. Type I is usually the mildest form, with normal or near-normal growth and minimal bone deformities. Type II is lethal in the perinatal period, with multiple intrauterine fractures and bone deformities of the extremities. Type III is the most severe form in children who survive the neonatal period, with fractures often presenting at birth. These children have extremely short stature with progressive limb and spine deformities secondary to multiple fractures. Type IV is the most phenotypically heterogeneous group, with mild to moderate bone deformities and variable short stature. In 2012, van Dijk et al. [6] reporting for the committee on best practice guidelines for diagnosis and investigation noted 8 types of OI based on clinical characteristics and molecular genetic defects. However, OMIM now records 17 OI types and a further 3 syndromic forms of OI have been defined at the molecular level, 2 types with craniosynostosis/skull deformity and one type with significant eye involvement.

To date, more than 1,000 different *COL1A1*/*COL1A2* mutations have been identified in patients with OI (https://oi.gene.le.ac.uk, accessed July 20, 2015). There are two general categories of type I collagen mutations giving rise to OI types I to IV. The first are haploinsufficiency mutations caused by frameshift, nonsense, and splice-site mutations, which lead to failure to synthesize the products of one *COL1A1* allele. The second involves the synthesis of collagen molecules with structural abnormalities, most frequently caused by the substitution of glycine by another amino acid in the Gly-X-Y triplet domain of the triple helix. Previous studies have reported that OI patients with *COL1A1* haploinsufficiency mutations have milder bone fragility and damage than those with *COL1A1*/*COL1A2* helical glycine mutations [7, 8]. Both *COL1A1* and *COL1A2* genes are very large, and they have rarely been analyzed systematically in Taiwan. The aim of this study was to characterize the correlations of genotype and phenotype for Taiwanese OI patients, with the hope that this may aid in the clinical diagnosis, genetic counseling and prenatal diagnosis of this disease.

## Patients and methods

### Study population

Seventy-two Taiwanese patients (27 males and 45 females; age range at last follow-up, 0.2-62 years) from 37 unrelated families were diagnosed with OI during the study period (January 1996 through December 2014) at Mackay Memorial Hospital, Taipei, Taiwan. None of the patients belonged to a consanguineous family. Molecular analysis was performed to investigate the presence of *COL1A1* or *COL1A2* mutations. This is an analysis of only those patients found to have a molecular genetic finding of a mutation in one of the two type I collagen genes. Clinical manifestations and the results of physical examinations and imaging studies of these patients were also recorded. The hospital’s Ethics Committee approved the study protocol, and all of the participants or their parents provided written informed consent.

### Clinical assessment

Diagnosis and classification were based on clinical and radiological characteristics according to the Sillence classification system [5]. In this study, every patient was evaluated at our clinic by one author, in person, to minimize inter-observer variation. For each patient, we recorded their height, weight, and bone mineral density (BMD) standard deviation scores (SDSs), walking ability and family history, and the occurrence of blue sclera, DI, hearing loss, bone deformity, and scoliosis. Height and weight were transformed to SDSs on the basis of a standard growth table for Taiwanese children and adolescents [9]. The BMD of the lumbar spine (L1–L4) was assessed using dual energy X-ray absorptiometry (DEXA) with a Hologic QDR 4500 system (Hologic, Bedford, MA, USA) [4]. The BMD results were converted to age- and gender-specific SDSs based on the normative reference data for BMD in Taiwanese children and adults [10, 11]. Pamidronate therapy has been reported to increase BMD, decrease the fracture rate, and substantially improve functional status for OI patients [12]. However, none of our patients had received pamidronate treatment at the time of assessment.

### Mutation analysis for type I collagen

Total genomic DNA was isolated from peripheral blood using standard extraction methods. DNA sequencing of all 51 polymerase chain reaction-amplified exons of the *COL1A1* gene and 52 exons of the *COL1A2* gene, including the intron-exon boundaries, was performed using a BigDye Terminator cycle sequencing kit (Applied Biosystems, Foster City, CA, USA). The nucleotide sequence was determined using an Applied Biosystems 3100 DNA sequencer. Sequence traces were aligned with the GenBank reference sequences of *COL1A1* genomic DNA (AF017178.2) and cDNA (NM_000088.3), and *COL1A2* genomic DNA (AF004877.1) and cDNA (NM_000089.3). DNA mutation numbering was based on the cDNA sequence using the A of the ATG translation initiation start site as nucleotide +1. Novel mutations were identified by their absence from the Osteogenesis Imperfecta Variant Database (https://oi.gene.le.ac.uk/home.php). In addition, identified nucleotide changes were re-examined in 100 control alleles. Polymorphisms were considered if the same nucleotide changes were detected in the control group.

### Statistical analysis

Relationships between gender, age, BMD SDS, DI and the existence of each clinical feature in the OI patients were tested using Pearson correlation, and significance was tested using Fisher *r*-*z* transformations. The clinical and radiological data were compared between patients with helical mutations versus those with haploinsufficiency mutations using the Student’s *t*-test for continuous variable, and Pearson’s chi-squared test and Fisher’s exact test for categorical variables. Two-tailed *p*-values were computed. SPSS version 11.5 (SPSS Inc., Chicago, IL) was used for calculations and differences were considered to be statistically significant when the *p* value was less than 0.05.

## Results

### Clinical characteristics

A review of the patients’ medical records revealed that among the 72 OI patients, 42 (58 %) were classified as OI type I, 5 (7 %) as type III, and 25 (35 %) as type IV. None of the patients had OI type II. The SDSs for height, weight, and BMD for all patients were–1.61 ± 2.57,–0.84 ± 1.56, and–2.06 ± 1.62, respectively. Furthermore, when dividing the patients into groups by OI type, the height SDSs were–0.49 ± 1.37,–6.42 ± 5.54, and–2.51 ± 1.85, respectively; and the BMD SDSs were–1.57 ± 1.15,–3.32 ± 1.86, and − 2.69 ± 1.96, respectively, for type I, III, and IV. A triangular face, blue sclera, DI, hearing loss, bone deformity, scoliosis, and walking without assistance were recorded in 18, 89, 43, 19, 58, 36 and 90 % of the patients, respectively. An older age was associated with hearing loss (*p* < 0.01). The BMD SDS was positively correlated with the ability to walk without assistance (*p* < 0.01). Patients with DI appeared to be prone to developing bone deformities (*p* < 0.01) and scoliosis (*p* < 0.01). Girls with OI had a slightly higher BMD SDS than boys with the same mutation type (*p* < 0.05) (Additional file 1: Table S1 and Additional file 2: Table S2).

### *COL1A1* and *COL1A2* mutations

Thirty-seven *COL1A1* and *COL1A2* mutations were identified in the 72 patients, including 28 *COL1A1* mutations and 9 *COL1A2* mutations. None of the *COL1A1* or *COL1A2* mutations were the same among these 37 unrelated families, and 15 (41 %) were novel mutations. Among the 28 *COL1A1* mutations, 7 were missense mutations, 4 were nonsense mutations, 6 were splicing mutations, and 11 were frameshift mutations. Eight familial mutations were identified. All 9 *COL1A2* mutations were missense mutations, 4 of which were from familial inheritance (Tables 1 and 2). Among the 37 causative *COL1A1* and *COL1A2* mutations, 15 (41 %) were caused by the substitution of a glycine within the Gly-X-Y triplet domain of the triple helix. There were 7 glycine mutations in *COL1A1* and 8 in *COL1A2*. Among the 15 glycine mutations, aspartic acid substitutions were the most common type (*n* = 6, 40 %), followed by serine (*n* = 4, 27 %), arginine (*n* = 2, 13 %), cysteine (*n* = 2, 13 %), and glutamic acid (*n* = 1, 7 %) substitutions. Of the 21 haploinsufficiency mutations in *COL1A1*, 11 were frameshift mutations, 6 were splicing mutations, and 4 were nonsense mutations.

**Table 1:** Genetic findings of 28 OI probands with mutations in *COL1A1*

Family No.	Type of OI	Exon or Intron	Nucleotide change (DNA level)	Predicted amino acid change (protein level)	Mutation type	Helical mutation or haploinsufficiency	Novel mutation	Familial/Sporadic
F1	I	Exon 4	c.333-9A > G		Splicing	Haploinsufficiency	Yes	S
F2	I	Exon 5	c.441delC		Frameshift	Haploinsufficiency		S
F3	I	Exon 5	c.386_387insC		Frameshift	Haploinsufficiency	Yes	F
F4	IV	Exon 5	c.391C > T	p. Arg131X	Nonsense	Haploinsufficiency		S
F5	IV	Exon 6	c.477_478 insT		Frameshift	Haploinsufficiency	Yes	S
F6	I	Exon 7	c.579delT	p. Pro193Profs*72	Frameshift	Haploinsufficiency		F
F7	IV	Exon 8	c.642 + 1G > A		Splicing	Haploinsufficiency		S
F8	I	Exon 8	c.590G > A	p. Gly197Asp	Missense	Helical		S
F9	I	Exon 9	c.658C > T	p. Arg220X	Nonsense	Haploinsufficiency		F
F10	IV	Exon 11	c.769G > A	p. Gly257Arg	Missense	Helical		F
F11	IV	Intron 12	c.858 + 24G > A		Splicing	Haploinsufficiency	Yes	S
F12	III	Exon 13	c.878G > A	p. Gly293Asp	Missense	Helical	Yes	S
F13	III	Exon 16	c.1021G > C	p. Gly341Arg	Missense	Helical	Yes	S
F14	IV	Intron 17	c.1155 + 3_1155 + 6del	c.1155 + 3_6delAAGT	Splicing	Haploinsufficiency		S
F15	I	Intron 20	c.1354-12G > A		Splicing	Haploinsufficiency		F
F16	I	Exon 21	c.1380delT		Frameshift	Haploinsufficiency		F
F17	IV	Exon 24	c.1667delC		Frameshift	Haploinsufficiency	Yes	S
F18	I	Exon 24	c.1615-1G > T		Splicing	Haploinsufficiency	Yes	S
F19	IV	Exon 35	c.2384-2394 del 11 mers		Frameshift	Haploinsufficiency	Yes	S
F20	IV	Exon 36	c.2461G > A	p. Gly821Ser	Missense	Helical		S
F21	I	Exon 37	c.2523delT		Frameshift	Haploinsufficiency		S
F22	I	Exon 38	c.2644C > T	p. Arg882X	Nonsense	Haploinsufficiency		F
F23	I	Exon 40	c.2775delT		Frameshift	Haploinsufficiency	Yes	S
F24	III	Exon 42	c.3064G > A	p. Gly1022Ser	Missense	Helical		S
F25	I	Exon 42	c.3076C > T	p. Arg1026X	Nonsense	Haploinsufficiency		F
F26	IV	Exon 44	c.3124_3134del11		Frameshift	Haploinsufficiency	Yes	S
F27	IV	Exon 47	c.3505G > A	p. Gly1169Ser	Missense	Helical		S
F28	III	Exon 52	c.4308_4309insA		Frameshift	Haploinsufficiency		S

*OI* osteogenesis imperfecta

**Table 2:** Genetic findings of 9 OI probands with mutations in *COL1A2*

Family No.	Type of OI	Exon or Intron	Nucleotide change (DNA level)	Predicted amino acid change (protein level)	Mutation type	Helical mutation or haploinsufficiency	Novel mutation	Familial/Sporadic
F29	I	Exon 8	c.335G > A	p. Gly112Asp	Missense	Helical	Yes	F
F30	IV	Exon 24	c.1378G > A	p. Gly460Ser	Missense	Helical		S
F31	IV	Exon 29	c.1666G > T	p. Gly556Cys	Missense	Helical	Yes	S
F32	IV	Exon 33	c.2018G > A	p. Gly673Asp	Missense	Helical		S
F33	I	Exon 37	c.2197G > T	p. Gly733Cys	Missense	Helical		F
F34	IV	Exon 37	c.2279G > A	p. Gly760Glu	Missense	Helical		F
F35	III	Exon 37	c.2288G > A	p. Gly763Asp	Missense	Helical		S
F36	I	Exon 40	c.2531G > A	p. Gly844Asp	Missense	Helical	Yes	F
F37	IV	Exon 51	c.3815G > C	p. Cys1272Ser	Missense	-	Yes	S

*OI* osteogenesis imperfecta

### Genotype and phenotype analysis

**Fig. 1:**
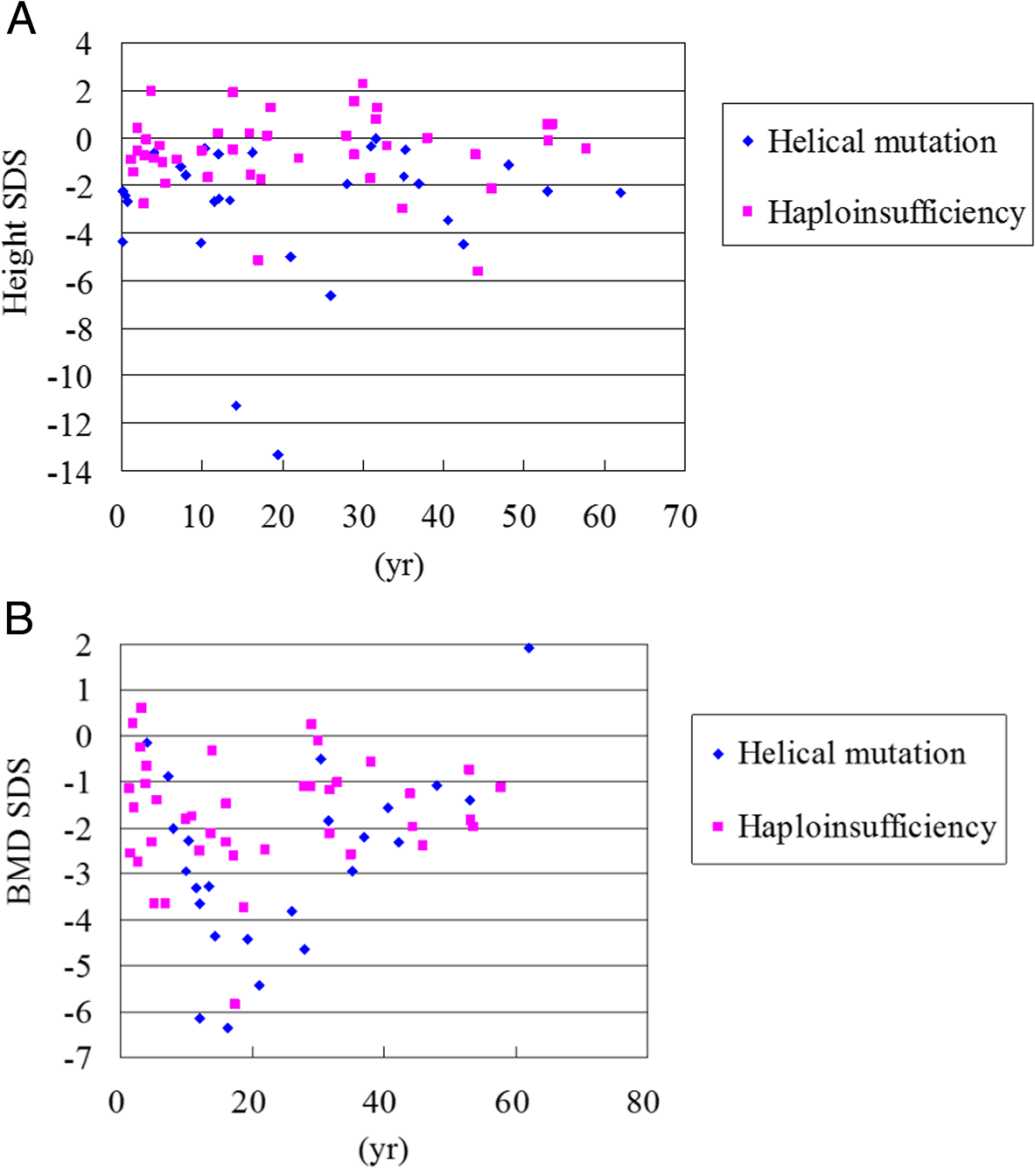
Relationships between age and **a** height SDS (*n* = 71), **b** BMD SDS (*n* = 64) of different mutation types [*COL1A1*/*COL1A2* helical glycine mutation (blue dot) vs. *COL1A1* haploinsufficiency mutation (pink dot)] in patients with osteogenesis imperfecta. SDS, standard deviation score; BMD, bone mineral density

The OI type was correlated with mutated genes and mutation types. Compared with *COL1A2* mutations, *COL1A1* mutations were more frequent in the patients with OI type I (46 % vs. 33 %) and III (14 % vs. 11 %), and less frequent in the patients with OI type IV (39 % vs. 56 %). Helical mutations were more frequent in the patients with OI type III (27 % vs. 5 %) and IV (47 % vs. 38 %), and less frequent in the patients with OI type I (27 % vs. 57 %) compared to those with haploinsufficiency mutations (Table 3). Among the 72 patients, 51 had a *COL1A1* mutation and 21 had a *COL1A2* mutation. Twenty-nine patients had helical mutations and 42 had haploinsufficiency mutations. Compared with haploinsufficiency, the patients with helical mutations had more severely damaged skeletal phenotypes, including shorter height, lower weight and BMD, poorer walking ability, and more frequent manifestations of DI and scoliosis (*p* < 0.05) (Table 4, Fig: 1a and b).

**Table 3:** The relationship between OI type and mutated genes and mutation types

Mutated genes and mutation types	OI type		
	I	III	IV
*COL1A1* (*n* = 28)	13 (46 %)	4 (14 %)	11 (39 %)
*COL1A2* (*n* = 9)	3 (33 %)	1 (11 %)	5 (56 %)
Helical mutation (*n* = 15)	4 (27 %)	4 (27 %)	7 (47 %)
Haploinsufficiency (*n* = 21)	12 (57 %)	1 (5 %)	8 (38 %)

*OI* osteogenesis imperfecta

**Table 4:** Relationship between clinical characteristics and different mutation types (helical mutation vs. haploinsufficiency) in *COL1A1* and *COL1A2* of 71 patients with OI at the time of bone densitometry analysis

	Helical mutation (*n* = 29)	Haploinsufficiency (*n* = 42)	*p* value
OI Type (I/III/IV)	9/4/16	33/1/8	<0.001
*COL1A1*/*COL1A2* mutation	9/20	42/0	<0.001
Gender (M/F)	12/17	15/27	0.635
Age (years)	21.8 ± 17.1	21.4 ± 17.0	0.941
Height SDS	−2.93 ± 3.04	−0.61 ± 1.61	<0.001
Weight SDS	−1.29 ± 1.67	−0.55 ± 1.44	<0.05
BMD SDS	−2.73 ± 1.96 (*n* = 24)	−1.70 ± 1.25 (*n* = 40)	<0.05
Triangular face	21 %	17 %	0.672
Blue sclera	83 %	95 %	0.085
Dentinogenesis imperfecta	62 %	31 %	<0.01
Hearing loss	17 %	21 %	0.668
Fracture at birth	14 %	5 %	0.184
Bone deformity	69 %	50 %	0.115
Scoliosis	52 %	26 %	<0.05
Walking without assistance	76 % (*n* = 25)	98 %	<0.01

*OI* osteogenesis imperfecta, *SDS* standard deviation score, *BMD* bone mineral density

## Discussion

A number of studies have described the clinical and molecular findings for Western patients with OI [13–17]; however, only a few studies have been conducted in Asian patients [8, 18, 19]. To the best of our knowledge, this is the first large-scale study to analyze the genotypes and phenotypes of Taiwanese patients with OI. We identified 37 different *COL1A1*/*COL1A2* mutations in 72 patients from 37 unrelated families, including 28 *COL1A1* mutations and 9 *COL1A2* mutations. Among these 37 *COL1A1*/*COL1A2* mutations, 16 (43 %) were missense mutations, 11 (30 %) were frameshift mutations, 6 (16 %) were splicing mutations, and 4 (11 %) were nonsense mutations. These mutations were unique and were not repeated between the families, meaning that there was no hot-spot *COL1A1*/*COL1A2* mutation in the study population. Fifteen (41 %) of the mutations were novel and 12 (32 %) were familial. In Zhang et al’s study [8], 19 of 58 (33 %) Chinese patients had familial OI, and Venturi et al. [17] reported that 7 of 22 (32 %) Italian OI patients were familial cases, both of which are consistent with our results. However, Lee et al. [18] described a higher incidence of familial OI with 18 of 34 (53 %) Korean patients. Further studies are needed to elucidate whether this is an artifactual difference related to a limitation in sample size or whether there are ethnic differences in the genetic defects underlying OI. Similar to previous population studies on OI in other countries [8, 14–19], most of our Taiwanese OI patients were categorized into type I (58 %), followed by type IV (35 %) and type III (7 %). In addition, none of the patients had OI type II in this study.

Growth retardation is a notable symptom of OI, and it is extremely severe in patients with OI type III. Moriwake and Seino [20] reported a national survey of Japanese OI patients with height SDSs of −3.36 ± 3.59,−7.83 ± 3.54, and − 4.63 ± 3.13 in patients with OI type I, III and IV, respectively, which is consistent with our findings.

Patients with OI have a significantly lower BMD. DEXA can detect a low BMD that may be missed on plain radiographs, even in patients with the milder forms of OI [21]. It may therefore aid in establishing the diagnosis, assessing the prognosis, and possibly monitoring the response to medical treatment. Among the 65 patients with available DEXA data of BMD in the present study, 94 % had a reduced BMD, 48 % with BMD SDS < −2, and 31 % with BMD SDS < −1 and ≧ − 2.

Blue sclera is a distinctive feature of unknown etiology in OI, and most patients with OI type I have blue sclera throughout their lives. In OI type III and IV, the sclera may also be blue at birth and during infancy, however the blue color fades with time during childhood [22]. The majority of our patients (89 %) had blue sclera.

DI is another principle manifestation of OI, and is associated with an abnormal type I collagen molecule. Discoloration and pulpal obliteration are the major characteristics. Lukinmaa et al. [23] reported that DI is frequently observed in OI type III and IV, but not commonly in type I. Our results were consistent with this finding, and 80 % of the OI type III patients had DI, followed by 56 % of the type IV patients and 31 % of the type I patients. We also found that the patients with DI appeared to be prone to developing bone deformities (*p* < 0.01) and scoliosis (*p* < 0.01).

Progressive hearing loss is an important symptom of OI, with the most common age at onset being in the second to fourth decades of life [24]. In our study, 19 % of the patients had hearing loss at a mean age of 39.1 years compared to 17.5 years without hearing loss, and an increase in age was associated with the presence of hearing loss (*p* < 0.01).

In patients with OI, the causes of bone deformities include an imperfect healing process after a fracture, and weight bearing itself without an apparent fracture. Bone deformities are frequently observed in the long bones, and scoliosis, spinal deformities, and compression fractures are also commonly seen [20]. In our study, 58 % of the patients presented with bone deformities.

The pathology of scoliosis is based on vertebral fragility, which progressively deteriorates with age [25]. Karbowski et al. [26] performed a nationwide cross-sectional study on German patients with OI, and reported that scoliosis was observed in 74.5 % (76/102) of their patients with an average age of 24.6 years. In our patients, 36 % (26/72) had scoliosis with a mean age of 27.3 years.

Rauch et al. [7] reported that girls with OI have a slightly higher BMD than boys with the same mutation type. Their histomorphometric observations also showed that bone formation and turnover rates were lower in girls than in boys. Lower bone turnover is expected to result in a slightly higher bone mass. The same results were also observed in our study.

There is a tendency to give more weighting to severity in phenotypic grouping whereas recent reviews stress that there is variability in severity encompassing mild to moderate phenotypes within families in patients with both OI type 1 and type 4 [3]. The persistence of deep blue-gray scleral hue should be given more weighting in favor of OI type 1 in families although scleral hue is difficult to assess in small children with OI type 4 who may have quite marked blue-gray sclera in infancy which subsequently fade to white in later childhood [22].

In our study cohort of Taiwanese OI patients, *COL1A1* mutations occurred 3.1 times (28:9) more frequently than *COL1A2* mutations, and the replacement of glycine residues by other residues within the Gly-X-Y triplet domain of the triple helix in both *COL1A1* and *COL1A2* was common. Our results are consistent with those of a previous study on Chinese patients [8]. Of the 15 glycine mutations, 6 (40 %) had glycine-to-aspartic acid substitutions, which is higher than previously reported in a mutation database (7.4 % in *COL1A1* and 15.8 % in *COL1A2*) [27]. The mutation database also reported that serine substitutions were the most common type of mutation in both *COL1A1* (38.9 %) and *COL1A2* (44 %) [27], however, only 4 (27 %) serine substitutions were identified in our sample.

We found that the OI type was correlated with mutated genes and mutation types. Rauch et al. [7] and Zhang et al. [8] both reported that compared with *COL1A2* mutations, *COL1A1* mutations were more frequent in patients with OI type I and less frequent in those with OI type IV. Compared with haploinsufficiency mutations, helical mutations occurred more commonly in the patients with OI type III and IV, and less commonly in the patients with OI type I. Although the Sillence classification is based exclusively on phenotypic criteria and is inevitably used inconsistently among different OI investigators, our results were still similar to previous reports [7, 8].

*COL1A1* haploinsufficiency mutations result in a quantitative defect, with synthesis of structurally normal type I procollagen at about half of the normal amount. They initiate nonsense-mediated decay of the mRNA derived from that allele leading to mild bone fragility. *COL1A1*/*COL1A2* helical glycine mutations cause the synthesis of collagen molecules with structural abnormalities, which can result in a clinical severity from mild OI type I to lethal OI type II. Previous studies have reported that OI patients with *COL1A1* haploinsufficiency mutations have milder bone fragility and damage than those with *COL1A1*/*COL1A2* helical glycine mutations [7, 8, 27], and this is consistent with our results.

### Limitations

Due to the limited sample size in this single-center retrospective study, substitutions by many amino acid residues as well as the position of a mutation occurred too infrequently to draw any conclusions about their phenotypic effects. In addition, our results were limited to BMD of the lumbar spine, which may not be representative of other skeletal involvement, especially the appendicular skeleton. The small sample size also reflects the rare nature of this genetic disorder, and both the age range and degree of disease severity varied widely. Therefore, further multicenter studies with larger cohorts and a longer follow-up period are warranted.

## Conclusion

Thirty-seven mutations were identified in the *COL1A1*/*COL1A2* genes and were associated with OI type I, III and IV by direct sequencing in our Taiwanese patients, including 28 *COL1A1* mutations and 9 *COL1A2* mutations. Among them, 15 (41 %) were novel mutations, 12 (32 %) were familial mutations, and 15 (41 %) were caused by the substitution of a glycine within the Gly-X-Y triplet domain of the triple helix. Patients with *COL1A1*/*COL1A2* helical glycine mutations had more severely damaged skeletal phenotypes than those with *COL1A1* haploinsufficiency mutations. Genotype and phenotype databases are expected to promote better genetic counseling and medical care of patients with OI.

### Ethics approval

Mackay Memorial Hospital IRB.

## Additional files

Additional file 1: Table S1. Clinical findings of 51 OI patients with mutations in *COL1A1* (*DOC 119 kb*) Additional file 2: Table S2. Clinical findings of 21 OI patients with mutations in *COL1A2* (*DOC 63 kb*)

## Abbreviations

BMD: bone mineral density
DEXA: dual energy X-ray absorptiometry
DI: dentinogenesis imperfecta
OI: osteogenesis imperfecta
SDS: standard deviation score
