# Supplementary material for: Genotype and phenotype analysis of Taiwanese patients with osteogenesis imperfecta
Source: Orphanet J Rare Dis. 2015 Dec 1;10:152. doi: 10.1186/s13023-015-0370-2 (PMC4666204; doi:10.1186/s13023-015-0370-2)
Supplement: Additional file 1: Table S1. — Clinical findings of 51 OI patients with mutations in COL1A1 (DOC 119 kb) [file 13023_2015_370_MOESM1_ESM.doc]

***Additional file 1: Table S1.*** *Clinical findings of 51 OI patients with mutations in COL1A1*

| Family No. | Patient No. | Gender | Age (years) | Type of OI | Height SDS | Weight SDS | BMD SDS | Triangular face | Blue sclera | DI | Hearing loss | Fractures at birth | Bone deformity | Scoliosis | Walking without assistance |
| --- | --- | --- | --- | --- | --- | --- | --- | --- | --- | --- | --- | --- | --- | --- | --- |
| F1 | P1 | F | 14 | I | 1.86 | 1.83 | -0.32 | + | + | - | - | - | - | - | + |
| F2 | P2 | F | 5.5 | I | -1.94 | -1.68 | -1.39 | - | + | - | - | - | - | - | + |
| F3 | P3 | M | 18 | I | 0.06 | 0.64 | NA | - | + | - | - | - | - | - | + |
|  | P4 | M | 19 | I | 1.23 | 0.64 | -3.72 | + | + | - | - | - | - | - | + |
|  | P5 | M | 53 | I | -0.15 | -0.70 | -1.83 | - | + | - | + | - | + | - | + |
| F4 | P6 | F | 58 | IV | -0.46 | -1.21 | -1.13 | + | + | + | + | - | + | + | + |
| F5 | P7 | M | 17 | IV | -1.76 | 0.41 | -5.84 | - | + | - | - | - | + | - | + |
| F6 | P8 | F | 2.1 | I | 0.43 | 1.62 | -1.58 | - | + | + | - | - | + | - | + |
|  | P9 | F | 3.8 | I | 1.93 | 0.33 | -1.05 | - | + | + | - | - | + | - | + |
|  | P10 | F | 16 | I | -1.55 | -1.74 | -2.32 | - | + | + | - | - | + | + | + |
|  | P11 | M | 22 | I | -0.89 | -1.68 | -2.47 | - | + | - | - | - | - | - | + |
|  | P12 | F | 29 | I | -0.71 | -0.55 | -1.11 | + | + | + | - | - | - | - | + |
|  | P13 | M | 30 | I | 2.26 | 2.56 | -0.12 | - | + | + | + | - | + | - | + |
|  | P14 | F | 32 | I | 0.77 | -0.94 | -1.17 | - | + | + | + | - | + | - | + |
|  | P15 | F | 53 | I | 0.56 | -1.34 | -0.75 | - | + | + | + | - | + | + | + |
|  | P16 | F | 54 | I | 0.56 | 0.74 | -1.98 | - | + | + | + | - | + | - | + |
| F7 | P17 | F | 3 | IV | -0.79 | -0.50 | -0.26 | - | + | - | - | - | - | - | + |
| F8 | P18 | F | 7.4 | I | -1.20 | -1.38 | -0.89 | - | + | - | - | - | + | + | - |
| F9 | P19 | F | 2 | I | -0.57 | -0.62 | 0.27 | - | + | - | - | - | - | - | + |
|  | P20 | M | 3.2 | I | -0.09 | -0.22 | 0.59 | - | + | - | - | - | - | - | + |
|  | P21 | F | 4 | I | -0.85 | -0.85 | -0.65 | - | + | - | - | - | - | - | + |
|  | P22 | F | 29 | I | 1.53 | 3.91 | 0.25 | - | + | - | + | - | - | - | + |
|  | P23 | F | 33 | I | -0.36 | -1.02 | -1.01 | - | + | - | - | - | + | - | + |
|  | P24 | M | 38 | I | 0.00 | -0.14 | -0.58 | - | + | - | + | - | - | - | + |
| F10 | P25 | F | 12 | IV | -2.67 | -1.22 | -3.31 | + | + | - | - | - | - | - | + |
|  | P26 | M | 13 | IV | -2.63 | -1.52 | -3.26 | + | + | - | - | - | - | - | + |
|  | P27 | M | 41 | IV | -3.44 | -2.12 | -1.58 | + | + | - | + | - | + | - | + |
| F11 | P28 | F | 44 | IV | -5.63 | -1.89 | -1.99 | - | - | - | - | - | + | + | + |
| F12 | P29 | F | 19 | III | -13.31 | -4.88 | -4.43 | - | + | + | - | + | + | + | - |
| F13 | P30 | M | 0.2 | III | -2.27 | -0.39 | NA | - | + | + | - | + | + | - | Undefined |
| F14 | P31 | F | 1.6 | IV | -1.44 | -2.25 | -2.55 | - | + | - | - | - | + | - | + |
| F15 | P32 | F | 10 | I | -0.55 | -0.89 | -1.81 | - | + | - | - | - | - | + | + |
|  | P33 | M | 12 | I | 0.19 | 0.62 | -2.49 | - | + | - | - | - | - | - | + |
|  | P34 | M | 44 | I | -0.73 | -1.92 | -1.25 | - | + | - | - | - | - | - | + |
| F16 | P35 | M | 7 | I | -0.95 | -1.28 | -3.65 | - | + | - | - | - | - | - | + |
|  | P36 | F | 16 | I | 0.20 | -0.46 | -1.48 | - | + | - | - | - | + | + | + |
|  | P37 | F | 28 | I | 0.10 | -0.02 | -1.10 | - | - | - | - | - | - | + | + |
| F17 | P38 | M | 5.2 | IV | -1.02 | 0.15 | -3.65 | - | + | - | - | - | + | - | + |
| F18 | P39 | M | 11 | I | -1.68 | -0.96 | -1.76 | + | + | + | - | - | + | - | + |
| F19 | P40 | F | 17 | IV | -5.14 | -2.44 | -2.60 | - | + | - | - | - | + | + | + |
| F20 | P41 | M | 21 | IV | -4.98 | -2.21 | -5.42 | + | - | + | - | - | - | + | - |
| F21 | P42 | F | 46 | I | -2.13 | -2.69 | -2.39 | - | + | - | - | - | + | + | + |
| F22 | P43 | M | 14 | I | -0.52 | -0.75 | -2.11 | - | + | + | - | - | - | - | + |
|  | P44 | F | 32 | I | 1.23 | -1.25 | -2.12 | + | + | + | - | + | + | + | + |
| F23 | P45 | F | 31 | I | -1.73 | -2.52 | NA | - | + | + | - | - | - | - | + |
| F24 | P46 | F | 0.2 | III | -4.35 | -0.68 | NA | - | + | + | - | + | + | + | Undefined |
| F25 | P47 | F | 4.8 | I | -0.32 | 1.68 | -2.31 | - | + | - | - | - | - | - | + |
|  | P48 | F | 35 | I | -2.98 | -2.68 | -2.57 | - | + | - | + | - | - | - | + |
| F26 | P49 | M | 2.8 | IV | -2.75 | -1.79 | -2.74 | + | + | - | - | - | + | - | + |
| F27 | P50 | M | 0.7 | IV | -2.65 | -2.25 | NA | - | + | + | - | - | + | - | Undefined |
| F28 | P51 | F | 1.2 | III | -0.90 | -1.14 | -1.17 | - | + | - | - | + | + | + | - |
| OI, osteogenesis imperfecta; SDS, standard deviation score; BMD, bone mineral density; DI, dentinogenesis imperfecta; NA, not available. | | | | | | | | | | | | | | | |
