# Supplementary material for: Genotype and phenotype analysis of Taiwanese patients with osteogenesis imperfecta
Source: Orphanet J Rare Dis. 2015 Dec 1;10:152. doi: 10.1186/s13023-015-0370-2 (PMC4666204; doi:10.1186/s13023-015-0370-2)
Supplement: Additional file 2: Table S2. — Clinical findings of 21 OI patients with mutations in COL1A2 (DOC 63 kb) [file 13023_2015_370_MOESM2_ESM.doc]

***Additional file 2: Table S2.*** *Clinical findings of 21 OI patients with mutations in* COL1A2

| Family No. | Patient No. | Gender | Age (years) | Type of OI | Height SDS | Weight SDS | BMD SDS | Triangular face | Blue sclera | DI | Hearing loss | Fractures at birth | Bone deformity | Scoliosis | Walking without assistance |
| --- | --- | --- | --- | --- | --- | --- | --- | --- | --- | --- | --- | --- | --- | --- | --- |
| F29 | P52 | M | 35 | I | -0.48 | -1.95 | -2.93 | + | + | + | - | - | - | - | + |
|  | P53 | F | 37 | I | -1.95 | -1.65 | -2.21 | - | + | - | - | - | + | + | + |
| F30 | P54 | F | 10 | IV | -4.42 | -2.74 | -2.95 | - | + | + | - | - | + | + | + |
| F31 | P55 | M | 8 | IV | -1.56 | 1.02 | -2.02 | - | + | + | - | - | + | - | + |
| F32 | P56 | M | 26 | IV | -6.62 | -1.60 | -3.82 | - | - | + | - | - | + | + | - |
| F33 | P57 | M | 4 | I | -0.60 | -0.43 | -0.15 | - | + | - | - | - | - | - | + |
|  | P58 | F | 31 | I | -0.36 | 0.17 | -0.51 | - | + | - | - | - | - | - | + |
|  | P59 | F | 35 | I | -1.61 | -1.89 | NA | - | - | - | - | - | - | - | + |
|  | P60 | M | 42 | I | -4.46 | -3.09 | -2.32 | - | - | - | - | - | + | - | + |
| F34 | P61 | F | 10 | IV | -0.46 | 1.70 | -2.29 | - | + | + | + | - | + | + | + |
|  | P62 | F | 12 | IV | -0.64 | -0.79 | -3.66 | - | + | + | - | - | + | + | + |
|  | P63 | F | 12 | IV | -2.54 | -0.69 | -6.15 | - | + | + | - | - | + | + | - |
|  | P64 | M | 16 | IV | -0.60 | 1.43 | -6.36 | - | - | + | - | - | + | + | + |
|  | P65 | M | 32 | IV | 0.00 | 0.94 | -1.83 | - | + | + | - | - | + | + | + |
|  | P66 | F | 48 | IV | -1.13 | -1.89 | -1.06 | - | + | + | - | - | + | + | + |
|  | P67 | F | 53 | IV | -2.26 | -0.69 | -1.40 | - | + | + | + | - | - | - | + |
|  | P68 | F | 62 | IV | -2.30 | 1.62 | 1.92 | + | + | + | + | - | + | + | + |
| F35 | P69 | F | 14 | III | -11.26 | -3.76 | -4.36 | - | + | + | - | + | + | + | - |
| F36 | P70 | F | 0.4 | I | -2.40 | -4.25 | NA | - | + | - | + | - | - | - | Undefined |
|  | P71 | F | 28 | I | -1.94 | -2.36 | -4.65 | - | + | - | - | - | + | - | + |
| F37 | P72 | F | 30 | IV | -4.91 | -0.30 | -0.51 | - | - | - | - | - | + | - | + |
| OI, osteogenesis imperfecta; SDS, standard deviation score; BMD, bone mineral density; DI, dentinogenesis imperfecta; NA, not available. | | | | | | | | | | | | | | | |
